# Predictors of Self-Determined Module Choice in a Web-Based Computer-Tailored Diet and Physical Activity Intervention: Secondary Analysis of Data From a Randomized Controlled Trial

**DOI:** 10.2196/15024

**Published:** 2020-07-23

**Authors:** Juul M J Coumans, Catherine A W Bolman, Anke Oenema, Lilian Lechner

**Affiliations:** 1 Department of Health Psychology Faculty of Psychology Open University of the Netherlands Heerlen Netherlands; 2 Department of Health Promotion Caphri, Faculty of Health, Medicine and Life Sciences Maastricht University Maastricht Netherlands

**Keywords:** eHealth, computer-tailoring, self-determination theory, multiple health behaviors, lifestyle promotion, module choice

## Abstract

**Background:**

Tailoring an online intervention to participant preferences (eg, by giving participants a choice which modules to follow) may increase engagement in the intervention, motivation for behavioral change, and possibly intervention effects. So far, little is known about what characteristics predict these module choices. Filling this knowledge gap is useful for optimizing program engagement.

**Objective:**

We investigated participant choice for a dietary and/or physical activity (PA) promotion module in our web-based computer-tailored intervention based on self-determination theory (SDT) and motivational interviewing (MI). Furthermore, we investigated which demographic characteristics, current behavior, psychosocial constructs and constructs from SDT and MI, and program-related variables such as advice on which module to follow were associated with these choices.

**Methods:**

Observational data were used from the randomized controlled trial MyLifestyleCoach of participants who were randomized into the intervention condition, completed the baseline questionnaire, and made a module choice in the opening session of the intervention. Here, they received advice on their own dietary and PA behavior. At the session’s end, they chose which lifestyle modules they would like to follow (both, diet, PA, or no module). Measurements included demographic information; self-reported diet and PA; and several psychosocial, SDT, and MI constructs. In total, data from 619 Dutch adults (59.6% women; mean age was 51.9 [SD 13.5] years) were analyzed. A stepwise multinomial logistic regression analysis was conducted to investigate which characteristics are related to module choice; the diet module served as reference category as almost everyone was advised to follow this module.

**Results:**

Of this sample, 54.8% (339/619) chose to do both the diet and PA module, 25.4% (157/619) chose to follow the diet module, 17.8% (110/619) preferred to follow no module, and 2.1% (13/619) chose to do the PA module only. Furthermore, it was found that older people, those who consumed more fruit, and those who scored lower on importance to change their current diet were more likely to choose no module compared to the diet module. People who had more motivation to change their current PA and those who received strong advice compared with slight advice to follow the diet module were more likely to choose both modules compared with the diet module only.

**Conclusions:**

The results show that more than half of the sample was interested in following both the diet and PA module in this online lifestyle intervention. Several characteristics were found to be related to module choice. A future challenge is to examine how this knowledge can be used to improve future interventions, such as tailoring (messages or content) on specific groups or examining where and how MI could be used to motivate people to make a certain module choice.

**Trial Registration:**

Netherlands Trial Register NL7333; https://www.trialregister.nl/trial/7333

## Introduction

Health risk behaviors such as unhealthy dietary intake and insufficient physical activity (PA) are widespread among adults, making the prevention of these behaviors a public health priority [1-3]. The small effect sizes, limited sustainability of effects, and high dropout rates usually found with existing online diet and PA interventions suggest that there is room for improvement in this field [4,5]. In this regard, there is more and more evidence showing the importance of autonomous motivation in sustained behavioral change [6,7], and this might therefore be a potential strategy for improving diet and PA interventions. Self-determination theory (SDT) postulates that providing conditions that support autonomy and choice are important basic psychological needs to improve more autonomous forms of motivation [8-11]. This autonomy support may be relevant during participation in intervention programs but also necessary at the start of an intervention, allowing participants the option to choose for themselves what parts of the intervention program they want to participate in. For interventions targeting multiple behaviors, this means people should have the option to choose which behavior they want to work on, preferably from separate modules so they can focus on a behavior in which they are really interested. Until now, research about the choices people make in such interventions (ie, which modules people choose) has been limited. But research that focuses on which factors and determinants are related to these choices is even more scarce.

To date, research has mainly investigated predictors of participation, use, and revisits of eHealth interventions instead of why people choose certain topics in these interventions [12-19]. In general, these studies have shown that women, older people, people with more education, people with a higher income, and people with a healthier BMI and a healthier lifestyle are more likely to visit, start, use, and revisit web-based interventions. In addition, insufficient time, no personalized content, dissatisfaction with the content, and computer difficulties were linked to a decrease in adherence [20]. Giving participants a choice of which modules to follow may improve user engagement and favorably affect compliance and long-term success [21]. One study found that a majority of patients showed improvements in anxiety and depressive symptoms when they could decide what modules to use in a tailored web-based treatment, and only one participant (of out 27) dropped out [22].

Yet only a few studies have described which behaviors (modules) individuals choose to start with in multiple health behaviors interventions [21]. Two studies in the general adult population found that people prefer to select the PA module over dietary modules [19,23]. In a study with cancer survivors, a reversed pattern was present: the diet module was preferred over the PA module [24]. Another study found no favorable effects of messages tailored to a participant-selected topic over an expert-determined topic on PA levels [25]. Based on these mixed findings, the role of module choice should be further explored in tailored web-based interventions.

So far, two studies have identified factors that relate to module choice in online interventions [23,24]. These studies have demonstrated that age, marital status, advice (both content and number of referrals), and healthier lifestyle have been related to module choice. Research to date has not yet determined whether psychosocial factors also relate to module choice. According to the SDT, individuals experience more autonomous forms of motivation when the needs for relatedness, autonomy, and competence are met [10,26]. Thus, feeling a higher degree of autonomy and perceived competence in changing or maintaining improved dietary intake or PA may lead to more intrinsic motivation to change behavior, which could subsequently lead to engagement in actions to achieve the intended behavior change [8-11]. The client-centered counseling style and techniques from motivational interviewing (MI) can create a facilitating environment in which these needs are promoted [27]. However, (intrinsic) motivation alone is often not sufficient for initiating and maintaining improved nutrition or PA levels. Other psychosocial characteristics closely linked to motivation may also play a role in this process and possibly in module choice. It may be that people who have a higher commitment to change certain behaviors are more likely to choose a certain module. However, to our knowledge, no study has examined to what extent these factors relate to module choice [28].

With eHealth interventions becoming more and more popular in recent years, there is now a general idea concerning elements that make an intervention effective and increase its use. However, a detailed understanding of what actually occurs within an intervention is still lacking. Our main aim is to elucidate factors related to module choice. Identifying and understanding which characteristics relate to the choice of a specific module or the choice not to follow any module can inform intervention improvement. This knowledge can be used to optimally tailor the intervention to the specific characteristics and needs of the participants and prevent nonuse. This may result in increasing intervention exposure and relevance that could result in increased effectiveness of the intervention.

In this study, participant choice of a dietary and/or PA promotion module is investigated in our web-based computer-tailored intervention based on SDT/MI. The main aim of this study is to examine which factors are related to these module choices (diet, PA, both, or no module) in the intervention. For these factors, we will look at demographic characteristics, psychosocial constructs related to SDT and MI, current behavior, and program-related variables (advice on which module to follow and the importance of changing behavior after feedback). Several hypotheses have been formulated based on previously mentioned literature. Regarding demographic characteristics, it is expected that women, older people, and people with more education are more inclined to choose a module (both, diet, PA) compared with no module. Furthermore, it is expected that a higher intrinsic motivation to perform a certain healthy behavior is related to choosing that particular module (ie, a higher motivation to engage in sufficient PA is related to choosing the PA or PA and diet module compared with the diet or no module). In addition, a healthier lifestyle is likely to affect module choice as well (eg, people with a healthier diet should be more inclined to choose the diet module compared with no module). Last, advice to follow a particular module and importance of the targeted change behavior is also expected to relate to choosing that particular module (both, diet, PA) compared with no module.

## Methods

### Design

This observational study was conducted as part of a 2-armed randomized controlled trial, MyLifestyleCoach. Data were collected in the period from mid-October 2018 until mid-May 2019. A comprehensive description of the intervention and the underlying theoretical frameworks, details of the trial design, and sample size calculations have been published elsewhere [29,30]. This web-based computer-tailored dietary and PA promotion intervention was developed using the intervention mapping protocol, and it is based on principles from SDT and MI. MyLifestyleCoach consists of two modules: I Move, an existing effective computer-tailored intervention to promote PA (PA module), and I Eat, which is aimed at promoting healthy eating (diet module), using similar behavior strategies as the I Move module [31]. The I Eat module is new, however, and its effectiveness has not been evaluated yet [29]. This study has been reviewed and approved by the Committee for Ethics and Consent in Research of the Open University of the Netherlands (reference number: U2018/07266/SVW) and was registered with the Netherlands Trial Register [NL7333].

### Participants

**Figure 1:**
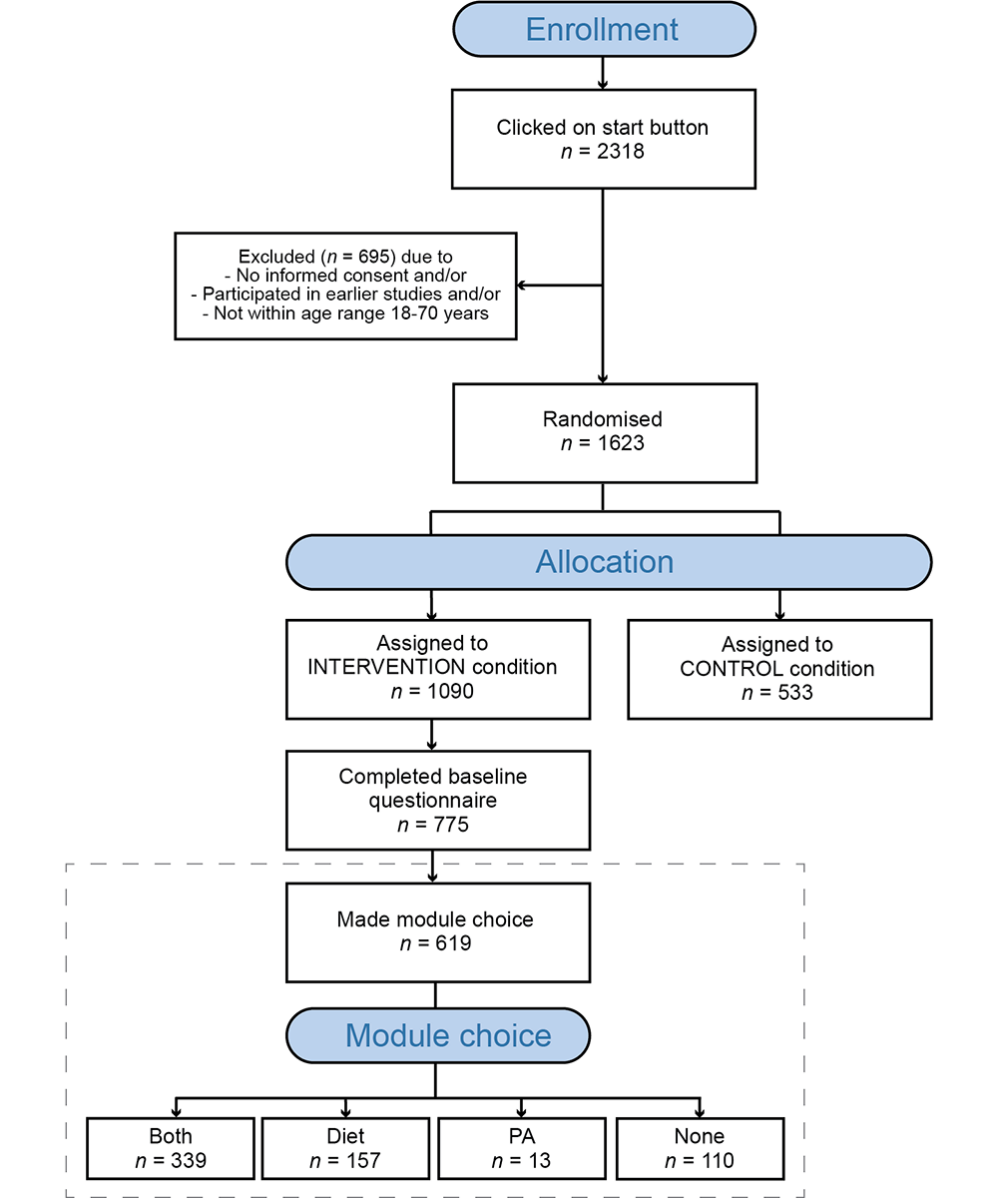
Overview of the study design.

Participants for this study were recruited via an internet research panel of Dutch inhabitants who occasionally volunteer in web-based research. They were eligible to participate if they were aged 18 to 70 years. A total of 1090 participants were randomized into the intervention condition of this study, of which 71.10% (775/1090) of participants completed the baseline questionnaire and 79.9% (619/775) of participants made a module choice in the opening session of MyLifestyleCoach. See Figure 1 for an overview of the study design and flow of participants. The dashed line represents the focus of this study.

### Measurements

All the following constructs were measured in the online self-reported baseline questionnaire except for perceived importance; this was measured at the end part of the opening session, after feedback regarding current behavior had been provided. Researchers had no influence on how participants reported the outcomes. Participants had to complete each question to be able to proceed to the next section of the online questionnaire.

#### Demographics

The baseline questionnaire assessed gender, age, educational level, marital status (with or without a partner), health status (on a scale from 0 to 100), and height and body weight to calculate BMI. Educational level (ie, the highest level of education completed) was recoded into three categories: low (primary or basic vocational school), medium (secondary vocational school or high school), and high (higher vocational school or university). Work was recoded into two categories: employed (full-time or part-time work) and unemployed (volunteering, having no job, incapacitated to work, retired, household chores, or studying). Furthermore, we asked whether participants had an impairment that would prevent them from being physically active (yes/no).

#### Psychosocial Constructs Including Self-Determination Theory/Motivational Interviewing–Related Constructs

##### Competence

Two Perceived Competence Scales were used to assess perceived competence (basic psychological need, SDT) for a healthy diet and PA [32]. This is a 4-item questionnaire on which participants indicated the extent to which each statement is true for them on a 7-point Likert scale (1 = not at all, 7 = very true). An example item is: “I feel confident in my ability to maintain a healthy diet/exercise regularly.” The perceived competence scores were determined by calculating a mean score for the 4 items, separately for diet and PA. The internal consistency of these scales in this study was excellent (Cronbach α=.95 for diet and .96 for PA).

##### Self-Regulation and Motivation

The Treatment Self-Regulation Questionnaire concerns why people would engage in health-relevant behavior [33,34]. It was used to assess the degree to which a person’s motivation for a healthy diet and PA is relatively driven by autonomous or self-determined reasons (SDT). Participants indicated the extent to which each of the 15 items was true for them on a 7-point Likert scale (1 = not at all, 7 = very true) for healthy eating and PA. Six items measure an autonomous regulatory style (Cronbach α=.92 for diet and .93 for PA), 6 items measure a controlled regulatory style (Cronbach α=.86 for diet and .89 for PA), and 3 items assess amotivation (Cronbach α=.70 for diet and .76 for PA). Responses to the respective items for each regulatory style were averaged to obtain a score for each of the three self-regulatory styles.

##### Intrinsic Motivation

According to the SDT, autonomous motivation consists of identified, integrated, and intrinsic regulation. However, the Treatment Self-Regulation Questionnaire does not differentiate between these autonomous forms of motivation. Therefore, the subscale intrinsic regulation from the Dutch Behavioral Regulation in Exercise Questionnaire was administered to determine intrinsic motivation, as this is the only fully self-determined form of motivation [35]. Participants rated on a 5-point Likert scale to what extent each of the 4 items was true for them with values of 1 = not true for me to 5 = very true to me. An example item was: “I exercise because it’s fun” (translated to engage in PA in Dutch). To compare intrinsic motivation for exercise with a healthy diet as closely as possible, we replaced exercise in all items by eating healthily. The scores of the 4 items were averaged into one intrinsic motivation score, both for a healthy diet and PA. The internal consistency of these scales in this study was excellent (Cronbach α=.93 for diet and .98 for PA).

##### Importance

In the opening session, participants were asked to rate their perceived importance of eating (more) healthily and becoming more physically active using an importance ruler after they received feedback on their current behavior. This is a 1 to 10 scale, a tool to support the use of MI [36]. Including this factor next to the baseline importance scale was necessary because importance could have been changed as a result of the content (eg, feedback on current behavior) of the opening session.

##### Intention

Three items were used to measure intention for eating healthier and being physically active [37]. Participants rated on a 10-point scale to what extent, how strong, and how probably they intended to eat (more) healthily and become physically more active (or stay sufficiently active). A mean score was calculated for diet and PA based on these 3 items. The internal consistency of these scales in this study was excellent (Cronbach α=.91 for diet and .95 for PA).

##### Commitment

Three items were used to measure commitment to eating healthier and being physically active [38]. Participants rated on a 5-point Likert scale how important eating (more) healthily/PA is for them, how involved they were in eating (more) healthily/PA, and how committed they are toward eating (more) healthily/PA. A mean score was calculated for diet and PA based on these items. The internal consistency of these scales in this study was good (Cronbach α=.89 for diet and .92 for PA).

#### Diet

Diet was assessed with a validated Food Frequency Questionnaire [39,40]. It assesses the frequency and quantity of a variety of food items eaten in a typical week in the last month. Participants reported on how many days they typically consumed fruit, vegetables, and fish (ranging from 0 to 7 days per week). We added questions assessing the size of vegetables and fruit portions based on Willems et al [41]. The intake of pieces of fruit per day was calculated by multiplying the frequency by the number of pieces with the reported number of consumption days, divided by 7 (days a week). When participants reported eating fruit or vegetables on at least one day but did not fill in the portion size, the portion size was replaced by the median. Furthermore, participants reported the consumption frequency of a particular snack food in a typical week in the last month on a 7-point Likert scale: 1 = never/less than once a week, 2 = 1 to 3 times a week, 3 = 4 to 6 times a week), 4 = 1 time per day, 5 = 2 times per day, 6 = 3 times per day, or 7 = 4 or more times per day. Eight types of snacks were assessed, namely unsalted nuts, dried fruits, chocolate, candy, cookies, chips, ice cream, and savory pastries. The consumption frequency of unhealthy snacks per day was determined by summing the recoded frequencies for last 6 snacks (chocolate to savory pastries) and dividing by the numbers of days in a week.

#### Physical Activity Level

PA behavior was assessed using the validated self-administered Dutch Short Questionnaire to Assess Health [42]. This questionnaire has been adapted to an online format. Participants were asked on how many days of in a typical week (0-7) during the past month they engaged in (1) walking to work/school, (2) cycling to work/school, (3) work, (4) household activities, (5) walking, (6) cycling, (7) gardening, and (8) odd jobs. If participants engaged at least once a week in the before mentioned activities, they reported how many hours and how many minutes they engaged in this activity. Work and household activities were separated for light/moderate and vigorous activity (examples were provided for light/moderate and vigorous activities). For sports, participants were asked which sports they engaged in the last month (they could choose up to 4 sports). Then they provided the number of days per week and duration for these sports. For walking, cycling, gardening, and sports, participants rated whether they considered it to be light, moderate, or vigorous. PA behavior was operationalized as the total number of minutes of moderate to vigorous physical activity per week (MVPA). This was calculated by multiplying the frequency (how many days per week), and duration (how many hours and minutes per day) of leisure and transport walking, leisure and transport cycling, gardening, household activities, odd jobs, and sports performed with moderate or vigorous intensity. Values were inspected using frequency tables, and extreme values (eg, 16 hours of heavy household activities) were replaced by the median of the sample. This self-report was used as this was the most feasible method (ie, convenience, low costs, and proven as a reliable and valid tool) in assessing PA compared with objective observations [43].

### Procedure

Members of the research panel received an email advertising the study about lifestyle containing information regarding the study (background, providing personal advice regarding dietary and PA behavior, that the program could help to improve their dietary and PA behavior, and information about prizes they could win for fully completing the intervention) and a link to the study website. Participation was free. When they clicked on the link, they could read further extensive information regarding the project, target population, expectations, data protection regulations, benefits (such as insight in current dietary and PA behavior and having a chance to win prizes), and contact details. If they were interested, they could click on the start button.

All eligible participants who agreed to participate answered some questions relevant to the inclusion and exclusion criteria and signed an online consent form. They were then computer randomized into the intervention condition or waiting list control condition (2:1) using a computer-determined sequence and completed the baseline questionnaire. In this paper, only respondents of the intervention condition were included in the analysis. After completing the baseline questionnaire, participants in the intervention condition continued directly to the opening session. Here, they were introduced to the structure of the program by text and by video coach. The video coach did not go into detail about the content of the modules or give advice on which module to follow. Participants were also asked to rate their perceived importance of eating (more) healthily and becoming more physically active on a 10-point scale. They received advice on which module to follow using a traffic light system based on the responses to the baseline questionnaire on where there was room for improvement, separately for diet and PA (see more information about this in the advice section). Participants were given a feedback message consisting of a summary of the extent to which they adhered to the diet and PA guidelines, in which domains there was room for improvement, and how important a healthy diet and sufficient PA was to them. They also received a brief reminder about the structure of the program.

Subsequently, participants were invited to choose which module they wanted to follow: diet, PA, both, or no module. The ones who chose no module received an email 2 weeks later with a link to the program where they could again make a module choice. Within the program, participants who had chosen to follow either the diet or the PA module were given the option the follow the other module once they had completed a session. Participants who did not immediately enter the opening session after completing the baseline questionnaire got a friendly reminder to do so 1 week after they completed the baseline questionnaire.

### Advice

**Figure 2:**
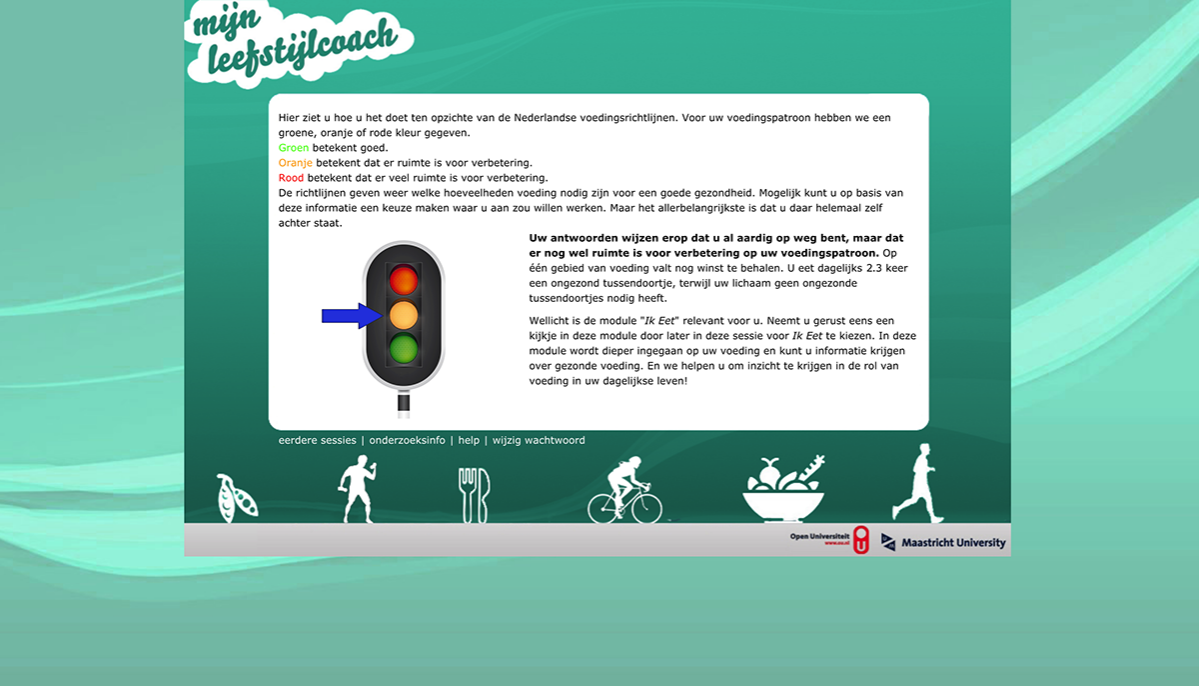
Screenshot of the page where people get advice in the opening session.

Participants received traffic light–based advice presented on two separate pages, one each for diet and PA. The color of the traffic light displayed in the advice indicated how closely their current PA level and diet corresponded to health recommendations. The corresponding text advised which modules were most relevant for them to use. For diet, the targeted behaviors (based on a pilot study) were to consume at least 200 grams of fruit (2 portions) and 250 grams of vegetables daily, eat fish at least once a week, and consume no unhealthy (ie, energy-dense) snacks [44]. For PA, the advice recommended accumulating at least 2.5 hours of MVPA every week [45]. A green light was shown when participants met the guideline. We then praised the positive scores and advised that it was not of high priority to follow the corresponding module but participants were still free to have a look at the module. An orange light was shown when the participants were close to meeting the guidelines (ie, the recommendations for at least one of the targeted behaviors for diet but not all were unmet or participant had 120 to 150 minutes of MVPA per week). Here, we also praised the reasonable positive score but advised following the module because there was room for improvement. Last, a red light was displayed when the participant failed to meet the guideline (ie, less than 120 minutes of MVPA per week). We strongly advised the participant to follow the corresponding module because the module could help them improve their behavior and health. If participants did not meet a certain guideline, they got detailed results about their current activity toward that particular behavior (fruit, vegetables, fish, unhealthy snacks, or PA) in relation to the guidelines. See Figure 2 for an example of the traffic light–based advice. The traffic lights were meant to provide participants with insight into their behavior and what they could change; they were not necessarily intended to induce compliance with dietary recommendations and PA guidelines.

### Statistical Analyses

Descriptive statistics were used to depict demographic and psychosocial characteristics of the participants included in this study. To investigate the predictors of module choice, we performed Spearman correlations between all predictors to determine the intercorrelation of predictors. In case of high intercorrelation, predictors could be removed beforehand. A stepwise multinomial logistic regression analysis was conducted to identify which demographic (step 1), psychosocial including SDT and MI constructs (step 2), dietary behavior (step 3), and program-related variables such as advice and changed importance (step 4) were related to module choice. Significance was set at *P*<.05. Multicollinearity was assessed using variance inflation factors (VIF). Furthermore, the fits of the models were compared with likelihood ratio tests. If *P*<.05, the more complex model was significantly better than the simpler model, and thus the more complex model was favored. All statistical analyses were performed with the statistical software R version 3.4.4 (R Foundation for Statistical Computing).

## Results

### Participants

The study population was slightly overrepresented by women (369/619, 59.6%; men: 250/619, 40.4%). The mean age was 51.9 years, and about two-thirds of the participants were in a relationship. Furthermore, the majority of the participants had a high level of education. Of this sample, more than half of the participants had a paid job (full-time or part-time). Of those who did not have a paid job, 8.9% (20/225) were volunteering, 7.1% (16/225) were unemployed, 16.4% (37/225) were incapacitated to work, 44.0% (99/225) were retired, 16.4% (37/225) were studying, and 7.1% (16/225) did household chores as their main activity. In total, 4.8% (30/619) of participants had a physical impairment that prevented them from being physically active. The mean BMI was 26.6 kg/m^2^; 1.8% (11/619) were classified as underweight, 42.2% (261/619) had a healthy weight, 35.2% (218/619) were classified as having nonobese overweight, and 20.8% (129/619) were classified as obese. About one in three participants complied with the fruit guideline of 2 portions of fruit per day, 11.8% (73/619) adhered to the vegetable guideline of 250 grams of vegetables per day, 62.5% (387/619) of this sample consumed fish at least once a week, and 7.8% (48/619) never consumed unhealthy snacks. More than 90% of this sample engaged in 150 minutes or more MVPA per week (Table 1).

**Table 1:** Characteristics of the study population.

Characteristics			Module choice				
			Both (n=339)	Diet (n=157)	PA^a^ (n=13)	None (n=110)	Total (n=619)
**Demographics**							
	>Age in years, mean (SD)		52.1 (12.8)	49.3 (15.1)	52.8 (14.3)	54.7 (12.6)	51.9 (13.5)
	**Gender, n (%)**						
		Women	215 (63.4)	97 (61.8)	7 (53.8)	50 (45.5)	369 (59.6)
		Men	124 (36.6)	60 (38.2)	6 (46.2)	60 (54.5)	250 (40.4)
	**Education, n (%)**						
		Low	13 (3.8)	6 (3.8)	1 (7.7)	4 (3.6)	24 (3.9)
		Medium	94 (27.7)	38 (24.2)	4 (30.8)	23 (20.9)	159 (25.7)
		High	232 (68.4)	113 (72.0)	8 (61.5)	83 (75.5)	436 (70.4)
	**Marital status, n (%)**						
		Partner	226 (66.7)	102 (65.0)	10 (76.9)	77 (70.0)	415 (67.0)
		Single	113 (33.3)	55 (35.0)	3 (23.1)	33 (30.0)	204 (33.0)
	**Employment status, n (%)**						
		Employed	210 (61.9)	104 (66.2)	7 (53.8)	73 (66.4)	394 (63.7)
		Unemployed	129 (38.1)	53 (33.8)	6 (46.2)	37 (33.6)	225 (36.3)
	**Physical impairment, n (%)**						
		No	322 (95.0)	147 (93.6)	13 (100)	107 (97.3)	589 (95.2)
		Yes	17 (5.0)	10 (6.4)	0 (0.0)	3 (2.7)	30 (4.8)
	**BMI group, n (%)**						
		Underweight	4 (1.2)	4 (2.5)	1 (7.7)	2 (1.8)	11 (1.8)
		Normal	137 (40.4)	63 (40.1)	4 (30.8)	57 (51.8)	261 (42.2)
		Overweight	112 (33.0)	62 (39.5)	5 (38.5)	39 (35.5)	218 (35.2)
		Obese	86 (25.4)	28 (17.8)	3 (23.1)	12 (10.9)	129 (20.8)
	BMI (kg/m^2^), mean (SD)		27.1 (5.7)	26.4 (5.0)	26.1 (4.7)	25.4 (3.6)	26.6 (5.2)
	Health status (1-100), mean (SD)		68.3 (16.2)	69.2 (16.9)	73.7 (9.0)	74.1 (13.7)	69.7 (16.0)
**Psychosocial and SDT^b^/MI^c^, mean (SD)**							
	**Competence (1-7)**						
		Diet	5.0 (1.5)	4.9 (1.3)	4.7 (1.4)	5.3 (1.4)	5.1 (1.4)
		PA	5.2 (1.5)	5.3 (1.3)	4.8 (1.7)	5.3 (1.5)	5.2 (1.4)
	**Amotivation (1-7)**						
		Diet	2.3 (1.2)	2.4 (1.2)	2.4 (1.3)	2.4 (1.2)	2.3 (1.2)
		PA	2.0 (1.2)	2.3 (1.3)	2.3 (1.5)	2.2 (1.2)	2.2 (1.2)
	**Controlled regulatory style (1-7)**						
		Diet	2.8 (1.3)	2.8 (1.1)	2.7 (1.3)	2.7 (1.1)	2.8 (1.2)
		PA	2.7 (1.3)	2.8 (1.2)	2.7 (1.2)	2.7 (1.1)	2.7 (1.2)
	**Autonomous regulatory style (1-7)**						
		Diet	5.5 (1.1)	5.4 (1.2)	5.1 (1.1)	5.4 (1.1)	5.5 (1.1)
		PA	5.7 (1.1)	5.5 (1.2)	5.5 (1.3)	5.6 (1.1)	5.6 (1.1)
	**Intrinsic motivation (1-5)**						
		Diet	3.4 (1.0)	3.4 (1.0)	3.1 (1.0)	3.5 (1.0)	3.4 (1.0)
		PA	3.8 (1.1)	3.9 (1.1)	3.6 (1.4)	3.9 (1.1)	3.8 (1.1)
	**Intention (1-10)**						
		Diet	7.9 (1.5)	7.7 (1.4)	7.5 (2.1)	7.9 (1.6)	7.8 (1.5)
		PA	7.8 (1.7)	7.9 (1.8)	7.0 (1.4)	7.7 (1.8)	7.8 (1.7)
	**Commitment (1-5)**						
		Diet	3.9 (0.6)	3.8 (0.7)	3.8 (0.7)	3.9 (0.6)	3.9 (0.7)
		PA	3.8 (0.8)	4.0 (0.8)	3.8 (0.8)	3.8 (0.8)	3.9 (0.8)
**Compliance to diet and PA guidelines, n (%)**							
	**Diet**						
		Fruit	106 (31.3)	50 (31.8)	3 (23.1)	52 (47.3)	211 (34.1)
		Vegetables^d^	36 (10.6)	16 (10.2)	1 (7.7)	12 (10.9)	65 (10.5)
		Fish	206 (60.8)	98 (62.4)	9 (69.2)	74 (67.3)	387 (62.5)
		Snacks	29 (8.6)	7 (4.5)	3 (23.1)	9 (8.2)	48 (7.8)
		Diet	3 (0.9)	0 (0.0)	0 (0.0)	0 (0.0)	3 (0.5)
	**PA**						
		MVPA^e,f^	314 (93.7)	152 (97.4)	9 (69.2)	104 (96.3)	579 (94.6)
**Program-related variables**							
	**Module advised, n (%)**						
		Both	21 (6.2)	4 (2.5)	5 (38.5)	4 (3.6)	34 (5.5)
		Diet	315 (92.9)	153 (97.5)	8 (61.5)	106 (96.4)	582 (94.0)
		PA	0 (0.0)	0 (0.0)	0 (0.0)	0 (0.0)	0 (0.0)
		None	3 (0.9)	0 (0.0)	0 (0.0)	0 (0.0)	3 (0.5)
	**Importance (1-10), mean (SD)**						
		Diet	8.2 (1.4)	8.0 (1.3)	7.8 (1.5)	7.9 (1.4)	8.1 (1.4)
		PA	8.5 (1.3)	8.4 (1.3)	8.5 (1.2)	8.4 (1.4)	8.5 (1.3)

^a^PA: physical activity.

^b^SDT: self-determination theory.

^c^MI: motivational interviewing.

^d^Vegetables: corrected values were used. Portion sizes of 75 grams or larger were divided by 50, as one portion equals 50 grams.

^e^MVPA: moderate to vigorous physical activity.

^f^Corrected values were used according to the Short Questionnaire to Assess Health manual; extreme values were furthermore replaced by the median. As a consequence, 7 participants were excluded as they reported to be physically active over 6720 minutes per week.

### Module Advice and Module Choice

Table 2 displays the provided module advice and subsequent module choice. In total, 24.6% (152/619) of participants were strongly advised to follow the diet module (red traffic light), 75.0% (459/619) of participants were slightly advised to follow the diet module (orange traffic light), and only 0.5% (3/619) of participants were not advised to follow the diet module (green traffic light). Regarding the PA advice, 3.6% (22/619) of participants were strongly advised to follow the PA module (red traffic light), 1.9% (12/619) of participants were slightly advised to follow the PA module (orange traffic light), and 94.5% (585/619) of participants were not advised to follow the PA module (green traffic light). Of the participants who made a module choice in the opening session of the intervention, 54.8% (339/619) of participants chose to follow both modules, 25.4% (157/619) of participants only chose the diet module, 2.1% (13/619) of participants chose only the PA module, and 17.8% (110/619) of participants chose no module. Thus, more than 80% of the participants were interested in following at least one module, and more than half of the participants were interested in following both modules.

**Table 2:** Overview of advice and module choice.

Traffic light^a^		Module choice					Total
Diet	PA^b^		Both, n (%)	Diet, n (%)	PA, n (%)	None, n (%)	N (%)
Red	Red		5 (1.5)	1 (0.6)	0 (0.0)	1 (0.9)	7 (1.1)
Red	Orange		2 (0.6)	0 (0.0)	1 (7.7)	0 (0.0)	3 (0.5)
Red	Green		86 (25.4)	39 (24.8)	1 (7.7)	16 (14.5)	142 (22.9)
Orange	Red		9 (2.7)	1 (0.6)	3 (23.1)	2 (1.8)	15 (2.4)
Orange	Orange		5 (1.5)	2 (1.3)	1 (7.7)	1 (0.9)	9 (1.5)
Orange	Green		229 (67.6)	114 (72.6)	7 (53.8)	90 (81.8)	440 (71.1)
Green	Red		0 (0.0)	0 (0.0)	0 (0.0)	0 (0.0)	0 (0.0)
Green	Orange		0 (0.0)	0 (0.0)	0 (0.0)	0 (0.0)	0 (0.0)
Green	Green		3 (0.9)	0 (0.0)	0 (0.0)	0 (0.0)	3 (0.5)
Total	—^c^		339 (54.8)	157 (25.4)	13 (2.1)	110 (17.8)	619 (100.0)

^a^Traffic light advice was based on self-reported (uncorrected) values. A red traffic light indicates that the module was strongly advised, an orange traffic light indicates that the module was slightly advised, and a green traffic light indicates that the module was not advised but that participants could have a look at the module.

^b^PA: physical activity.

^c^Not applicable

### Which Factors Are Related to Module Choice?

First, it was investigated using Spearman correlations whether there was collinearity between the continuous predictors. There were several predictors that were highly intercorrelated (*r*≥.60) with the other predictors. See Multimedia Appendix 1 for the correlation table. Based on these results, it was decided to remove the psychosocial constructs of intention, as the correlation coefficients were approaching a correlation coefficient of .80 with perceived competence (intention diet with perceived competence of diet, *r*s=.78, *P*<.001, and intention PA with perceived competence of PA, *r*s=.76, *P*<.001) and because perceived competence was of more theoretical relevance in this study (basic psychological need in the SDT).

Since only 13 participants chose the PA module only, the frequency was too low to enable group comparisons and coefficients could not be reliably estimated. We, therefore, removed those cases from the analyses. Then the stepwise multinomial logistic regression analysis was conducted with module choice as the outcome (both modules, diet, or no module). The diet module served as the reference category, because everyone was advised to follow the diet module, except for 3 participants who were excluded here. Multimedia Appendix 2 presents the results of this analysis. The means each predictor presented in Table 1, stratified for module choice, may aid understanding.

Model 1 shows that people with a higher age were more likely to choose both modules as well as no module compared with the diet module; however, the effect that older adults were more likely to choose both modules became nonsignificant. When the psychosocial and SDT/MI predictors were added to the previous (model 2), it was found that people who had higher levels of amotivation to change their current PA levels were less likely to choose both modules compared with the diet module. Model 3 additionally shows that people who consumed more fruit per day were more likely to choose no module compared with the diet module. When the program-related variables (model 4) were added to model 3, it could be seen that people who were strongly advised to follow the diet module were more likely to choose both modules than those who were slightly advised compared with the diet module. Last, people who found it more important to change their current diet were less likely to choose no module compared with the diet module.

### Model Comparisons

Adding the predictors to each subsequent model led to an increased Nagelkerke *R*^2^. Models 2 and 3 did not have a significantly improved fit compared with model 1 (see Multimedia Appendix 3). However, model 4 had a significantly better fit than models 2 and 3, whereas compared with model 1 there was found a marginal statistically significant effect. Therefore, model 4 seems to explain our data best, as this model had a significantly better fit than the other models (except for model 1) and also was indicated by the highest Nagelkerke explained variance (see Multimedia Appendix 2).

### Additional Information Regarding the Regression Analysis

A VIF larger than 10 is an indication of multicollinearity (see Multimedia Appendix 4). We removed autonomous motivation and commitment for diet and PA from the analysis. Importance was nevertheless included in the analysis, as this turned out to be an important factor in relation to module choice. The results of the full model, including those predictors that were excluded because of a too large VIF, can be seen in Multimedia Appendix 5. Although minor differences in the results were found, our main findings seem to hold.

## Discussion

### Principal Findings

This study investigated participant choice for a dietary and/or PA promotion module in a web-based computer-tailored intervention based on SDT/MI. The main aim was to examine which factors were associated with these module choices. The results showed that more than half of the sample (55%) chose to follow both the diet and the PA module. About a quarter of the sample chose to follow to diet module only, 18% of the participants did not want to follow any module, and 2% chose the PA module only.

Our findings are in line with a previous study in which more participants used the diet module (58%) than the PA module (22%) [24]. But our findings are not consistent with several other studies in which the PA module was preferred over dietary modules that focused on limiting fat intake and increasing fruit and vegetable consumption [19,23,25]. Several explanations could be offered for this discrepancy. First, improvements in diet seemed to be far more necessary in our sample than improvements in PA levels. In our study, almost no one completely adhered to the dietary guidelines, whereas most participants already adhered to the MVPA guideline, which may be an overestimation of findings in the general Dutch population [46]. Second, people were completely free to decide for themselves which behavior they wanted to work on (ie, improving or maintaining current behavior) rather than simply trying to achieve or maintain the recommended levels as in line with Kanera and colleagues [41]. This may also be evident for a large proportion of the participants who chose both modules even though they were not advised to do so, indicating that for them it is important to maintain a healthy lifestyle. In other studies, there were restrictions in making a choice, such as only choosing modules when participants received orange or red traffic light advice [23]. Third, in our study, as well as in the study by Kanera and colleagues [41], the diet module was rather broad targeting multiple dietary behaviors; in other studies, individuals could choose more specific topics (eg, fat) which might be more relevant for some participants. Still, there were 110 participants in our study who were not interested in following either the diet or PA module, even though all participants were advised to choose at least the diet module. This is interesting since most studies did not investigate the explicit option of following no module, even though it is an important choice that might be common in the intervention practice and research.

The main aim of this study was to examine why some people are likely to make a particular module choice. Our results showed that age was the only demographic factor related to module choice. Older people were more likely to choose no module compared with the diet module. This is somewhat in contrast to our expectations and previous findings in which older people generally participated more in eHealth interventions in terms of start and (re)visit [14,15,17,19].

Surprisingly, amotivation was the only motivational construct found to be related to module choice, while we expected that especially intrinsic motivation was related to module choice. People who lacked motivation to become more physically active were less likely to choose both modules compared with the diet module alone, possibly because many already adhered to the MVPA guidelines and only those who were more motivated (ie, having lower amotivation scores for PA) were interested in the PA module. Next to these findings, it could still be that there are other motivational constructs that are more important in explaining module use, adherence/dropout, and behavioral change [6]; these will be investigated in a future study.

Previous research found that an already healthier lifestyle was related to module choice [23,24]. In our study, we only found that people who consumed more fruit per day were more likely to choose no module compared with the diet module. Almost half of the people who preferred no module already ate enough fruit according to the fruit consumption guideline of two servings per day. Sufficient fruit intake might be a reason why they were less interested in following the diet module. Eating behavior is very complex, and when people estimate how healthy their current diet is, they might use various indicators and fruit consumption may be an important one.

As expected, dietary advice did affect the modules people in our study chose. Participants who were strongly advised compared with those who were slightly advised to choose the diet module were more likely to choose both modules. It could be that these participants felt more need to change their lifestyle at a broader level. PA advice, on the other hand, did not affect module choice. These findings somehow validate our nondirectional goal of advice; many participants already adhered to the PA guideline but still preferred to choose the PA module in addition to the diet module. With regard to participant perceptions of how important they find eating healthy and getting sufficient PA, we found that people who considered it more important to eat more healthily during the opening session were less likely to choose no module compared with the diet module. To note, importance was measured after advice including feedback about their current behavior was given. It is conceivable that in this basic opening session awareness about their own unhealthy behavior was increased. As a result, people might have changed their perceptions on the importance of their dietary behavior. So in terms of the transtheoretical model of behavioral change, people who were in the precontemplation stage (ie, having no intention to change behavior) could have moved toward the contemplation stage (ie, intending to start the healthy behavior) as a result of the opening session [47]. It should further be noted that this model (including the program-related variables) had the highest explained variance and the best fit, meaning that compared with all other models we tested, this model explained our observed data the best.

By giving individuals the opportunity to not follow any of the offered modules—in accordance with SDT and MI principles [48,49]—we definitely lost people before the more specific parts of the intervention started. It is possible these participants were mainly interested in completing the questionnaires (eg, for the possibility of winning rewards). This may have resulted in additional dropout. However, participants received reminders in a later stage that they could still begin using the modules if they wanted, which has not been accounted for in this study. It would be interesting to see if the option of choosing no module would lead to less dropout during the actual modules, as it is expected that only motivated participants are actually participating. It could also be that for those who chose to follow no module, the opening session provided sufficient information to fulfill their needs (for example, by receiving feedback on their PA and dietary behavior) and possibly even change their health behavior.

The findings of this study are preliminary, making it difficult to provide concrete recommendations for intervention development. Several factors were identified that were linked to choosing no module instead of the advised diet module: an older age, eating more fruit, and finding eating healthily less important. More research is needed to find out why those individuals are more likely to choose no module compared with the diet module, thus examining what could be improved (ie, more relevant content, better tailoring to specific groups, using MI to improve importance as early as possible) to make them more likely to choose the diet module. Future research is also necessary to investigate how and when MI could be applied to target those individuals who are more likely to choose no module and for whom the opening session is not sufficient to get them engaged in intervention activities and behavioral change.

### Strengths and Limitations

To our knowledge, this is the ﬁrst study to investigate to what extent the combined demographic characteristics, SDT/MI constructs, psychosocial factors, diet-related behavioral outcomes, and program-related variables (eg, advice) are related to participant module choice in a web-based tailored eHealth lifestyle intervention that is directed to more than one behavior. The findings of this study should be interpreted in the light of several limitations. First, we only used self-report to gather data. For example, even though the Dutch Short Questionnaire to Assess Health was the most feasible method to measure PA levels in our study, it may be subject to recall bias. In our study, a larger proportion of the people adhered to the PA guidelines compared with the general Dutch population, which could also be an overestimation of their actual PA level [46]. Second, our findings may not be fully generalizable to the entire Dutch adult population. A large part of our sample was highly educated. Even though our results showed that education did not affect module choice, generalizability may be questioned as a large proportion of the sample also adhered to the PA guideline, which may also concern overrepresentation [50]. Furthermore, women and people who are classified as obese were overrepresented as well, but age seems to be in line with the Dutch population. Third, attrition bias could play a role in several phases of the intervention period, such as between completing the baseline questionnaire but not completing the opening session (ie, making a module choice); this may threaten external and internal validity. This study did not provide insight into this matter; however, the process evaluation and effectiveness study will. Fourth, it may be that a combination of predictors and/or other predictors that are not measured in this study are also relevant in predicting module choice. No interaction effects were tested in this study since our sample size was not sufficient to test these effects on top of the already numerous predictors in our current analysis. Interactions between advice and the different types of motivation would be an interesting avenue to explore—for example, one can expect that people who score higher on controlled motivation might be more likely to follow advice within the intervention. Future research is necessary to examine additional relevant combinations of factors that could provide further insight into what predicts module choice.

### Conclusions

Most participants chose to follow both the diet and PA modules in our web-based tailored lifestyle intervention. Our study has provided new insights into which characteristics are related to module choice in a lifestyle intervention. Additional research is necessary to examine how to target those individuals who are likely to choose no module and thereby who are at risk of dropping out in studies where this option is not explicitly offered. Most of the SDT/MI–related concepts did not affect module choice, suggesting that for initial module choice constructs from the SDT were less relevant than expected. These factors may, nevertheless, be relevant at a later stage of the intervention, and they could be related to participation or behavioral change. This will be examined in a follow-up study. A stepped approach to develop and pilot an intervention may be of relevance as various factors may be related to different aspects of an intervention (ie, module choice, participation, and behavioral change). To conclude, the findings in this study have important implications for developing eHealth interventions containing multiple health behaviors, as they can provide input for intervention improvement by providing insights that could help to optimally tailoring to the needs and characteristics of participants, such as making it more interesting for older adults, increase its effectiveness, and prevent dropout in a later stage.

## Appendix

Multimedia Appendix 1 Correlation matrix for predictors.

Multimedia Appendix 2 Results from the stepwise multinomial logistic regression analysis predicting module choice.

Multimedia Appendix 3 Results from the model comparisons using likelihood ratio tests.

Multimedia Appendix 4 Variance inflation factors for each predictor in the stepwise multinomial regression analysis.

Multimedia Appendix 5 Results from the stepwise multinomial regression analysis using the full model.

## Abbreviations

MI: motivational interviewing
MVPA: moderate to vigorous physical activity
PA: physical activity
SDT: self-determination theory
VIF: variance inflation factor
