# Supplementary material for: Predictors of Self-Determined Module Choice in a Web-Based Computer-Tailored Diet and Physical Activity Intervention: Secondary Analysis of Data From a Randomized Controlled Trial
Source: J Med Internet Res. 2020 Jul 23;22(7):e15024. doi: 10.2196/15024 (PMC7413275; doi:10.2196/15024)
Supplement: Multimedia Appendix 1 [file jmir_v22i7e15024_app1.docx]

Multimedia Appendix 1. Correlation matrix for predictors

|  | 1 | 2 | 3 | 4 | 5 | 6 | 7 | 8 | 9 | 10 | 11 | 12 | 13 | 14 | 15 | 16 | 17 | 18 | 19 | 20 | 21 | 22 | 23 |
| --- | --- | --- | --- | --- | --- | --- | --- | --- | --- | --- | --- | --- | --- | --- | --- | --- | --- | --- | --- | --- | --- | --- | --- |
| 1. Age |  |  |  |  |  |  |  |  |  |  |  |  |  |  |  |  |  |  |  |  |  |  |  |
| 2. BMI | 0.20^***^ |  |  |  |  |  |  |  |  |  |  |  |  |  |  |  |  |  |  |  |  |  |  |
| 3. Health status | 0.17^***^ | -0.26^***^ |  |  |  |  |  |  |  |  |  |  |  |  |  |  |  |  |  |  |  |  |  |
| 4. Perc. comp. diet | 0.16^***^ | -0.23^***^ | 0.30^***^ |  |  |  |  |  |  |  |  |  |  |  |  |  |  |  |  |  |  |  |  |
| 5. Perc. comp. PA | 0.06 | -0.17^***^ | 0.40^***^ | 0.47^***^ |  |  |  |  |  |  |  |  |  |  |  |  |  |  |  |  |  |  |  |
| 6. Amot. diet | 0.01 | 0.18^***^ | -0.06 | -0.29^***^ | -0.23^***^ |  |  |  |  |  |  |  |  |  |  |  |  |  |  |  |  |  |  |
| 7. Amot. PA | 0.07 | 0.14^***^ | -0.02 | -0.17^***^ | -0.23^***^ | 0.70^***^ |  |  |  |  |  |  |  |  |  |  |  |  |  |  |  |  |  |
| 8. Contr. mot. diet | -0.01 | 0.07 | -0.06 | -0.04 | -0.01 | 0.24^***^ | 0.26^***^ |  |  |  |  |  |  |  |  |  |  |  |  |  |  |  |  |
| 9. Contr. mot. PA | -0.07 | -0.03 | 0.01 | -0.03 | 0.05 | 0.17^***^ | 0.25^***^ | 0.76^***^ |  |  |  |  |  |  |  |  |  |  |  |  |  |  |  |
| 10. Auton. mot. diet | 0.10^**^ | -0.07 | 0.12^**^ | 0.57^***^ | 0.38^***^ | -0.41^***^ | -0.28^***^ | 0.18^***^ | 0.14^***^ |  |  |  |  |  |  |  |  |  |  |  |  |  |  |
| 11. Auton. mot. PA | 0.13^***^ | -0.12^**^ | 0.16^***^ | 0.51^***^ | 0.56^***^ | -0.40^***^ | -0.35^***^ | 0.11^**^ | 0.16^***^ | 0.79^***^ |  |  |  |  |  |  |  |  |  |  |  |  |  |
| 12. IM diet | 0.08 | -0.26^***^ | 0.22^***^ | 0.60^***^ | 0.32^***^ | -0.28^***^ | -0.16^***^ | 0.11^**^ | 0.14^***^ | 0.57^***^ | 0.50^***^ |  |  |  |  |  |  |  |  |  |  |  |  |
| 13. IM PA | 0.06 | -0.25^***^ | 0.34^***^ | 0.38^***^ | 0.65^***^ | -0.17^***^ | -0.17^***^ | 0.002 | 0.07 | 0.33^***^ | 0.52^***^ | 0.41^***^ |  |  |  |  |  |  |  |  |  |  |  |
| 14. Comm. diet | 0.06 | -0.15^***^ | 0.15^***^ | 0.56^***^ | 0.26^***^ | -0.43^***^ | -0.27^***^ | 0.14^***^ | 0.09^*^ | 0.65^***^ | 0.51^***^ | 0.57^***^ | 0.26^***^ |  |  |  |  |  |  |  |  |  |  |
| 15. Comm. PA | 0.05 | -0.21^***^ | 0.29^***^ | 0.35^***^ | 0.59^***^ | -0.21^***^ | -0.23^***^ | 0.02 | 0.11^**^ | 0.35^***^ | 0.56^***^ | 0.31^***^ | 0.72^***^ | 0.33^***^ |  |  |  |  |  |  |  |  |  |
| 16. Intention diet | 0.12^**^ | -0.19^***^ | 0.29^***^ | 0.78^***^ | 0.43^***^ | -0.41^***^ | -0.28^***^ | -0.001 | 0.01 | 0.64^***^ | 0.56^***^ | 0.57^***^ | 0.36^***^ | 0.64^***^ | 0.34^***^ |  |  |  |  |  |  |  |  |
| 17. Intention PA | 0.07 | -0.14^***^ | 0.33^***^ | 0.44^***^ | 0.76^***^ | -0.25^***^ | -0.26^***^ | -0.01 | 0.08 | 0.40^***^ | 0.56^***^ | 0.29^***^ | 0.63^***^ | 0.28^***^ | 0.64^***^ | 0.51^***^ |  |  |  |  |  |  |  |
| 18. Fruit | 0.12^**^ | -0.07 | 0.15^***^ | 0.30^***^ | 0.18^***^ | -0.11^**^ | -0.09^*^ | -0.02 | -0.04 | 0.28^***^ | 0.20^***^ | 0.31^***^ | 0.23^***^ | 0.33^***^ | 0.18^***^ | 0.28^***^ | 0.19^***^ |  |  |  |  |  |  |
| 19. Vegetables^a^ | 0.06 | -0.06 | 0.15^***^ | 0.29^***^ | 0.09^*^ | -0.19^***^ | -0.20^***^ | 0.02 | 0.01 | 0.26^***^ | 0.23^***^ | 0.33^***^ | 0.12^**^ | 0.36^***^ | 0.13^**^ | 0.33^***^ | 0.14^***^ | 0.27^***^ |  |  |  |  |  |
| 20. Fish | 0.29^***^ | -0.02 | 0.19^***^ | 0.16^***^ | 0.14^***^ | -0.08^*^ | -0.05 | -0.04 | -0.03 | 0.12^**^ | 0.18^***^ | 0.13^**^ | 0.16^***^ | 0.14^***^ | 0.12^**^ | 0.15^***^ | 0.14^***^ | 0.18^***^ | 0.14^***^ |  |  |  |  |
| 21. Snacks | -0.10^*^ | -0.01 | -0.11^**^ | -0.27^***^ | -0.11^**^ | 0.10^*^ | 0.06 | 0.05 | 0.07 | -0.19^***^ | -0.15^***^ | -0.20^***^ | -0.12^**^ | -0.24^***^ | -0.11^**^ | -0.29^***^ | -0.11^**^ | -0.12^**^ | -0.16^***^ | -0.08^*^ |  |  |  |
| 22. MVPA^a^ | 0.11^**^ | -0.09^*^ | 0.24^***^ | 0.21^***^ | 0.28^***^ | 0.03 | 0.03 | 0.03 | 0.02 | 0.09^*^ | 0.11^**^ | 0.17^***^ | 0.32^***^ | 0.10^*^ | 0.33^***^ | 0.18^***^ | 0.29^***^ | 0.09^*^ | 0.09^*^ | 0.14^***^ | -0.11^**^ |  |  |
| 23. Importance diet | 0.08^*^ | -0.09^*^ | 0.18^***^ | 0.49^***^ | 0.36^***^ | -0.39^***^ | -0.28^***^ | 0.12^**^ | 0.08 | 0.67^***^ | 0.61^***^ | 0.48^***^ | 0.34^***^ | 0.67^***^ | 0.38^***^ | 0.67^***^ | 0.42^***^ | 0.22^***^ | 0.30^***^ | 0.13^***^ | -0.18^***^ | 0.13^**^ |  |
| 24. Importance PA | 0.12^**^ | -0.15^***^ | 0.26^***^ | 0.35^***^ | 0.57^***^ | -0.29^***^ | -0.29^***^ | 0.08 | 0.11^**^ | 0.48^***^ | 0.65^***^ | 0.32^***^ | 0.59^***^ | 0.38^***^ | 0.65^***^ | 0.49^***^ | 0.67^***^ | 0.14^***^ | 0.15^***^ | 0.14^***^ | -0.11^**^ | 0.25^***^ | 0.63^***^ |

*Note*. *P* ≤ .001 ‘^***^’, *P* ≤ .01 ‘^**^’, *P* ≤ .05 ‘^*^’; Grey shaded cells represent significance after Bonferroni correction. BMI = body mass index, Perc. comp. = perceived competence, PA = physical activity, amot = amotivation, auton = autonomous, contr. = controlled, mot = motivation, IM = intrinsic motivation, comm. = commitment.

^a^ Corrected values were used
