# Supplementary material for: Predictors of Self-Determined Module Choice in a Web-Based Computer-Tailored Diet and Physical Activity Intervention: Secondary Analysis of Data From a Randomized Controlled Trial
Source: J Med Internet Res. 2020 Jul 23;22(7):e15024. doi: 10.2196/15024 (PMC7413275; doi:10.2196/15024)
Supplement: Multimedia Appendix 2 [file jmir_v22i7e15024_app2.docx]

Multimedia Appendix 2. Results from the stepwise multinomial logistic regression analysis predicting module choice (*n* = 603)

|  | Module choice (reference category: diet module) | | | | | | | | | | | | | | | |
| --- | --- | --- | --- | --- | --- | --- | --- | --- | --- | --- | --- | --- | --- | --- | --- | --- |
|  | Model 1 | | | | Model 2 | | | | Model 3 | | | | Model 4 | | | |
|  | Both | | None | | Both | | None | | Both | | None | | Both | | None | |
| *Model 1: demographics* | OR | SE | OR | SE | OR | SE | OR | SE | OR | SE | OR | SE | OR | SE | OR | SE |
| Intercept | 1.12 | 0.83 | 0.24 | 1.19 | 1.47 | 1.02 | 0.32 | 1.40 | 1.74 | 1.03 | 0.48 | 1.44 | 1.01 | 1.43 | 0.41 | 2.13 |
| Age | 1.02^*^ | 0.01 | 1.03^**^ | 0.01 | 1.01^†^ | 0.01 | 1.03^**^ | 0.01 | 1.01 | 0.01 | 1.03^*^ | 0.01 | 1.02^†^ | 0.01 | 1.03^*^ | 0.01 |
| Sex^a^ | 0.88 | 0.21 | 1.59^†^ | 0.26 | 0.94 | 0.22 | 1.58^†^ | 0.27 | 0.96 | 0.22 | 1.60^†^ | 0.28 | 0.96 | 0.22 | 1.53 | 0.28 |
| Education high^b^ | 0.93 | 0.23 | 1.06 | 0.32 | 0.83 | 0.24 | 1.05 | 0.32 | 0.81 | 0.25 | 1.04 | 0.33 | 0.80 | 0.25 | 1.02 | 0.34 |
| Education low^b^ | 0.93 | 0.54 | 1.23 | 0.72 | 1.07 | 0.57 | 1.27 | 0.74 | 1.13 | 0.58 | 1.37 | 0.76 | 1.31 | 0.59 | 1.34 | 0.78 |
| Marital status^c^ | 1.00 | 0.22 | 0.91 | 0.29 | 0.99 | 0.22 | 0.94 | 0.29 | 0.99 | 0.22 | 0.95 | 0.30 | 1.01 | 0.23 | 0.97 | 0.30 |
| Employment^d^ | 0.84 | 0.22 | 1.09 | 0.29 | 0.82 | 0.22 | 1.07 | 0.29 | 0.81 | 0.22 | 0.98 | 0.29 | 0.84 | 0.22 | 1.004 | 0.30 |
| Physical impairment^e^ | 0.57 | 0.44 | 0.43 | 0.70 | 0.50 | 0.46 | 0.36 | 0.73 | 0.49 | 0.47 | 0.37 | 0.74 | 0.39^†^ | 0.49 | 0.32 | 0.77 |
| BMI | 1.02 | 0.02 | 0.94^†^ | 0.03 | 1.02 | 0.02 | 0.94^†^ | 0.03 | 1.02 | 0.02 | 0.94^†^ | 0.03 | 1.02 | 0.02 | 0.95 | 0.03 |
| Health status | 0.995 | 0.01 | 1.01 | 0.01 | 0.999 | 0.01 | 1.02 | 0.01 | 0.999 | 0.01 | 1.01 | 0.01 | 1.001 | 0.01 | 1.02^†^ | 0.01 |
|  |  |  |  |  |  |  |  |  |  |  |  |  |  |  |  |  |
| *Model 2: model 1 + psychosocial constructs* |  |  |  |  |  |  |  |  |  |  |  |  |  |  |  |  |
| Competence diet |  |  |  |  | 1.07 | 0.10 | 1.19 | 0.13 | 1.06 | 0.10 | 1.15 | 0.13 | 1.04 | 0.10 | 1.25 | 0.14 |
| Competence PA |  |  |  |  | 0.95 | 0.11 | 0.88 | 0.14 | 0.95 | 0.11 | 0.89 | 0.14 | 0.97 | 0.11 | 0.88 | 0.15 |
| Amotivation diet |  |  |  |  | 1.05 | 0.12 | 1.05 | 0.15 | 1.05 | 0.12 | 1.04 | 0.15 | 1.08 | 0.12 | 0.97 | 0.16 |
| Amotivation PA |  |  |  |  | 0.74^*^ | 0.12 | 0.90 | 0.15 | 0.74^*^ | 0.12 | 0.90 | 0.15 | 0.72^**^ | 0.12 | 0.92 | 0.15 |
| Controlled regulatory style diet |  |  |  |  | 1.14 | 0.14 | 1.09 | 0.18 | 1.12 | 0.14 | 1.06 | 0.18 | 1.10 | 0.15 | 1.15 | 0.19 |
| Controlled regulatory style PA |  |  |  |  | 0.97 | 0.14 | 0.95 | 0.18 | 0.997 | 0.14 | 0.99 | 0.18 | 0.995 | 0.14 | 0.94 | 0.19 |
| Intrinsic motivation diet |  |  |  |  | 1.07 | 0.14 | 1.04 | 0.18 | 1.05 | 0.14 | 0.99 | 0.19 | 1.03 | 0.15 | 1.08 | 0.19 |
| Intrinsic motivation PA |  |  |  |  | 0.87 | 0.13 | 0.81 | 0.17 | 0.85 | 0.13 | 0.77 | 0.17 | 0.86 | 0.14 | 0.75 | 0.19 |
|  |  |  |  |  |  |  |  |  |  |  |  |  |  |  |  |  |
| *Model 3: model 2 + dietary outcomes* |  |  |  |  |  |  |  |  |  |  |  |  |  |  |  |  |
| Fruit (portions/day) |  |  |  |  |  |  |  |  | 1.08 | 0.12 | 1.43^*^ | 0.14 | 1.18 | 0.12 | 1.49^**^ | 0.15 |
| Vegetables (grams/day) |  |  |  |  |  |  |  |  | 1.0001 | 0.001 | 0.999 | 0.002 | 1.0004 | 0.001 | 1.0001 | 0.002 |
| Fish (portions/week) |  |  |  |  |  |  |  |  | 0.98 | 0.10 | 0.997 | 0.12 | 1.11 | 0.12 | 1.05 | 0.15 |
| Unhealthy snacks (freq/day) |  |  |  |  |  |  |  |  | 0.94 | 0.06 | 0.94 | 0.08 | 0.94 | 0.06 | 0.92 | 0.08 |
|  |  |  |  |  |  |  |  |  |  |  |  |  |  |  |  |  |
| *Model 4: model 3 + program variables* |  |  |  |  |  |  |  |  |  |  |  |  |  |  |  |  |
| Advice diet (red vs. orange) |  |  |  |  |  |  |  |  |  |  |  |  | 1.85^*^ | 0.31 | 1.11 | 0.43 |
| Advice PA (green vs. orange) |  |  |  |  |  |  |  |  |  |  |  |  | 0.59 | 0.84 | 1.35 | 1.33 |
| Advice PA (red vs. orange) |  |  |  |  |  |  |  |  |  |  |  |  | 2.80 | 1.14 | 7.16 | 1.63 |
| Importance diet after feedback |  |  |  |  |  |  |  |  |  |  |  |  | 1.08 | 0.12 | 0.70^*^ | 0.15 |
| Importance PA after feedback |  |  |  |  |  |  |  |  |  |  |  |  | 0.98 | 0.13 | 1.20 | 0.17 |
|  |  |  |  |  |  |  |  |  |  |  |  |  |  |  |  |  |
| R^2^ Nagelkerke | 0.074 | | | | 0.105 | | | | 0.122 | | | | 0.156 | | | |

*Note. P* < .001 ‘^***^’, *P* < .01 ‘^**^’, *P* < .05 ‘^*^’, *P* < .1 ‘^†^’. Bold values indicate statistical significance at a significance level of α = 0.05. PA = physical activity; freq = frequency; OR = odds ratio, SE = standard error. MVPA and the dummy variable for green dietary advice versus orange were not added as predictors in the model as this led too high VIFs, and consequently, to an unstable model with uninterpretable predictors.

^a^ The group of women was the reference category.

^b^ Medium education served as the reference category.

^c^ The group of people without a relationship served as the reference category.

^d^ Having no paid job was the reference category

^e^ Having no physical impairment was the reference category
