# Supplementary material for: Predictors of Self-Determined Module Choice in a Web-Based Computer-Tailored Diet and Physical Activity Intervention: Secondary Analysis of Data From a Randomized Controlled Trial
Source: J Med Internet Res. 2020 Jul 23;22(7):e15024. doi: 10.2196/15024 (PMC7413275; doi:10.2196/15024)
Supplement: Multimedia Appendix 3 [file jmir_v22i7e15024_app3.docx]

Multimedia Appendix 3. Results from the model comparisons using likelihood ratio tests

| Model | Residual df | Residual Deviance | Test | Df | LR stat. | Pr(Chi) |
| --- | --- | --- | --- | --- | --- | --- |
| 1 | 1186 | 1050.07 |  |  |  |  |
| 2 | 1170 | 1132.56 | 1 vs 2 | 16 | 17.51 | 0.35 |
| 3 | 1162 | 1123.14 | 1 vs 3 | 24 | 26.93 | 0.31 |
|  |  |  | 2 vs 3 | 8 | 9.42 | 0.31 |
| 4 | 1152 | 1102.77 | 1 vs 4 | 34 | 47.30 | 0.06 |
|  |  |  | 2 vs 4 | 18 | 29.80 | 0.04 |
|  |  |  | 3 vs 4 | 10 | 20.37 | 0.03 |

*Note.* DF = degrees of freedom; LR = likelihood-ratio; Stat. = statistic; Pr(Chi) = *p* value from the Chi-Squared test.
