# Supplementary material for: Predictors of Self-Determined Module Choice in a Web-Based Computer-Tailored Diet and Physical Activity Intervention: Secondary Analysis of Data From a Randomized Controlled Trial
Source: J Med Internet Res. 2020 Jul 23;22(7):e15024. doi: 10.2196/15024 (PMC7413275; doi:10.2196/15024)
Supplement: Multimedia Appendix 4 [file jmir_v22i7e15024_app4.docx]

Multimedia Appendix 4. Variance Inflation Factors for each predictor in the stepwise multinomial regression analysis

|  | Model 1 | | Model 2 | | Model 3 | | Model 4 | |
| --- | --- | --- | --- | --- | --- | --- | --- | --- |
|  | Full | End | Full | End | Full | End | Full | End |
| Age | 5.08 | 5.08 | 5.39 | 5.26 | 5.56 | 5.43 | 5.59 | 5.47 |
| Sex | 1.62 | 1.62 | 1.69 | 1.67 | 1.70 | 1.67 | 1.70 | 1.68 |
| Education | 1.70 | 1.70 | 1.77 | 1.75 | 1.80 | 1.78 | 1.80 | 1.79 |
| Marital status | 2.16 | 2.16 | 2.22 | 2.18 | 2.22 | 2.18 | 2.22 | 2.19 |
| Employment status | 2.08 | 2.08 | 2.15 | 2.12 | 2.16 | 2.13 | 2.16 | 2.13 |
| Impairment | 1.19 | 1.19 | 1.24 | 1.23 | 1.25 | 1.24 | 1.26 | 1.26 |
| BMI | 6.62 | 6.62 | 7.17 | 7.01 | 7.15 | 6.99 | 7.15 | 7.02 |
| Health status | 5.88 | 5.88 | 6.37 | 6.27 | 6.42 | 6.32 | 6.57 | 6.45 |
|  |  |  |  |  |  |  |  |  |
| Perceived competence diet |  |  | 6.64 | 6.07 | 6.64 | 6.15 | 6.68 | 6.33 |
| Perceived competence PA |  |  | 7.43 | 6.83 | 7.45 | 6.85 | 7.61 | 7.20 |
| Amotivation diet |  |  | 3.91 | 3.68 | 3.95 | 3.71 | 4.00 | 3.85 |
| Amotivation PA |  |  | 3.60 | 3.48 | 3.67 | 3.55 | 3.71 | 3.61 |
| Controlled reg. style diet |  |  | 5.37 | 5.08 | 5.42 | 5.16 | 5.43 | 5.26 |
| Controlled reg. style PA |  |  | 5.12 | 4.92 | 5.16 | 4.99 | 5.18 | 5.00 |
| Autonomous reg. style diet |  |  | 11.48 |  | 12.00 |  | 12.27 |  |
| Autonomous reg. style PA |  |  | 12.10 |  | 12.51 |  | 13.11 |  |
| Intrinsic motivation diet |  |  | 6.37 | 5.89 | 6.44 | 6.01 | 6.51 | 6.16 |
| Intrinsic motivation PA |  |  | 7.40 | 6.20 | 7.42 | 6.24 | 7.53 | 6.67 |
| Commitment diet |  |  | 10.52 |  | 10.90 |  | 11.76 |  |
| Commitment PA |  |  | 9.83 |  | 9.97 |  | 10.46 |  |
|  |  |  |  |  |  |  |  |  |
| Fruit |  |  |  |  | 2.36 | 2.31 | 2.53 | 2.48 |
| Vegetables |  |  |  |  | 2.75 | 2.72 | 2.81 | 2.78 |
| Fish |  |  |  |  | 1.79 | 1.76 | 2.12 | 2.09 |
| Unhealthy snacks |  |  |  |  | 1.53 | 1.51 | 1.53 | 1.51 |
|  |  |  |  |  |  |  |  |  |
| Advice diet |  |  |  |  |  |  | 1.80 | 1.78 |
| Advice PA |  |  |  |  |  |  | 3.86 | 3.83 |
| Importance diet |  |  |  |  |  |  | 12.93 | 11.45^a^ |
| Importance PA |  |  |  |  |  |  | 14.32 | 13.08^a^ |

*Note*. BMI = body mass index; PA = physical activity; reg = regulatory.

Due to theoretical relevance it was decided to keep these concepts in the analysis.
